# Advancing Global Health through Development and Clinical Trials Partnerships: A Randomized, Placebo-Controlled, Double-Blind Assessment of Safety, Tolerability, and Immunogenicity of PfSPZ Vaccine for Malaria in Healthy Equatoguinean Men

**DOI:** 10.4269/ajtmh.17-0449

**Published:** 2017-10-30

**Authors:** Ally Olotu, Vicente Urbano, Ali Hamad, Martin Eka, Mwajuma Chemba, Elizabeth Nyakarungu, Jose Raso, Esther Eburi, Dolores O. Mandumbi, Dianna Hergott, Carl D. Maas, Mitoha O. Ayekaba, Diosdado N. Milang, Matilde R. Rivas, Tobias Schindler, Oscar M. Embon, Adam J. Ruben, Elizabeth Saverino, Yonas Abebe, Natasha KC, Eric R. James, Tooba Murshedkar, Anita Manoj, Sumana Chakravarty, Minglin Li, Matthew Adams, Christopher Schwabe, J. Luis Segura, Claudia Daubenberger, Marcel Tanner, Thomas L. Richie, Peter F. Billingsley, B. Kim Lee Sim, Salim Abdulla, Stephen L. Hoffman

**Affiliations:** 1 Ifakara Health Institute, Dar es Salaam, Tanzania;; 2 Equatorial Guinea Malaria Vaccine Initiative, Malabo, Equatorial Guinea;; 3 Ministry of Health and Social Welfare, Malabo, Bioko Norte, Equatorial Guinea;; 4 Medical Care Development International, Silver Spring, Maryland;; 5 Marathon EG Production Ltd, Punta Europa, Bioko Norte, Malabo, Equatorial Guinea;; 6 Swiss Tropical and Public Health Institute, Basel, Switzerland;; 7 La Paz Medical Center, Sipopo, Bioko Island, Equatorial Guinea;; 8 Sanaria Inc., Rockville, Maryland;; 9 Division of Malaria Research, Institute for Global Health, University of Maryland School of Medicine, Baltimore, Maryland;; 10 University of Basel, Basel, Switzerland

## Abstract

Equatorial Guinea (EG) has implemented a successful malaria control program on Bioko Island. A highly effective vaccine would be an ideal complement to this effort and could lead to halting transmission and eliminating malaria. Sanaria^®^ PfSPZ Vaccine (*Plasmodium falciparum* sporozoite Vaccine) is being developed for this purpose. To begin the process of establishing the efficacy of and implementing a PfSPZ Vaccine mass vaccination program in EG, we decided to conduct a series of clinical trials of PfSPZ Vaccine on Bioko Island. Because no clinical trial had ever been conducted in EG, we first successfully established the ethical, regulatory, quality, and clinical foundation for conducting trials. We now report the safety, tolerability, and immunogenicity results of the first clinical trial in the history of the country. Thirty adult males were randomized in the ratio 2:1 to receive three doses of 2.7 × 10^5^ PfSPZ of PfSPZ Vaccine (*N* = 20) or normal saline placebo (*N* = 10) by direct venous inoculation at 8-week intervals. The vaccine was safe and well tolerated. Seventy percent, 65%, and 45% of vaccinees developed antibodies to *Plasmodium falciparum* (Pf) circumsporozoite protein (PfCSP) by enzyme-linked immunosorbent assay, PfSPZ by automated immunofluorescence assay, and PfSPZ by inhibition of sporozoite invasion assay, respectively. Antibody responses were significantly lower than responses in U.S. adults who received the same dosage regimen, but not significantly different than responses in young adult Malians. Based on these results, a clinical trial enrolling 135 subjects aged 6 months to 65 years has been initiated in EG; it includes PfSPZ Vaccine and first assessment in Africa of PfSPZ-CVac. ClinicalTrials.gov identifier: NCT02418962.

## INTRODUCTION

Malaria has major public health significance in sub-Saharan Africa.^1^ World Health Organization-Global Technical Strategy has set a goal of eliminating malaria from at least 10 malaria-endemic countries by the year 2020.^2^ Development and deployment of new tools such as highly efficacious malaria vaccines that can interrupt malaria transmission will be essential to achieve this goal. The RTS,S/AS01 candidate malaria vaccine has shown moderate efficacy against clinical disease in young children,^3^ but its limited efficacy against infection and significant adverse events (AEs) restrict its usefulness for malaria elimination.^4,5^

Sanaria^®^ PfSPZ Vaccine is a live attenuated *Plasmodium falciparum* (Pf) whole sporozoite (SPZ) vaccine that is currently being assessed in clinical trials in the United States, Europe, and Africa. PfSPZ Vaccine has been well tolerated, safe, and protective against controlled human malaria infection (CHMI) in the United States and against natural exposure to malaria in Mali.^6,7^ Protection durable for at least 6–14 months has been demonstrated.^7–10^

A robust malaria control program has been in place on Bioko Island, Equatorial Guinea (EG) since 2004.^11^ Using insecticide-treated bed nets, indoor residual spraying and early diagnosis and treatment with artemisinin combination therapy,^11^ the Bioko Island Malaria Control Program has reduced the average prevalence of malaria parasitaemia in the island’s children from 45% in 2000 to 11.1% in 2016. To capitalize on this success, the EG Malaria Vaccine Initiative (EGMVI) was established in 2014 to assess the utility of PfSPZ Vaccine to eliminate malaria. EGMVI is funded by the Government of EG and three U.S. energy companies, Marathon EG Production Limited, Noble Energy, Atlantic Methanol Production Company, and EG LNG, and aims to culminate its activities with an island-wide mass vaccination program.

Here we report the findings from the first clinical trial in the history of EG. This was a phase 1 clinical trial to evaluate the safety, tolerability, and immunogenicity of three doses of 2.7 × 10^5^ PfSPZ of PfSPZ Vaccine administered by direct venous inoculation (DVI) to healthy, malaria-exposed semi-immune young Equatoguinean men living in a malaria-endemic region of Bioko Island, EG.

## MATERIALS AND METHODS

### Clinical trial design.

The primary objective of this study was to assess the safety, tolerability and immunogenicity of three doses of 2.7 × 10^5^ PfSPZ of PfSPZ Vaccine as compared with normal saline (NS) placebo administered to healthy young Equatoguinean men by DVI at 8-week intervals. The study was conducted at La Paz Medical Center, Malabo, EG. A Safety Monitoring Committee (SMC) was appointed by the study sponsor and consisted of three external experts and the local safety monitor, who was an independent Equatoguinean physician based in Malabo and accessible during the entire study period.

The study had three groups with a total of 33 volunteers. Group 1, the sentinel safety group, included three volunteers who received 1.35 × 10^5^ PfSPZ followed by 2.7 × 10^5^ PfSPZ of PfSPZ Vaccine 2 weeks later. The SMC reviewed the data from Group 1 and then recommended proceeding with vaccination of the rest of the volunteers.

For Groups 2 and 3, the study was a phase 1 randomized, double-blind, placebo-controlled clinical trial. Twenty volunteers in Group 2 received three doses of 2.7 × 10^5^ PfSPZ of PfSPZ Vaccine and 10 volunteers in Group 3 received three doses of NS at 8-week intervals.

### Ethical considerations.

Ethical approval was obtained from the National Ethics Committee of EG, the Ifakara Health Institute Institutional Review Board, Dar es Salaam, Tanzania, the MaGil Institutional Review Board in Maryland, USA, and the Ethical Review Committee of Northwestern and Central Switzerland. The study was registered at ClinicalTrials.gov (ClinicalTrials.gov registration number: NCT02418962). The volunteers were briefed on the specifics of the planned study, and then were assessed for their understanding of study procedures using a multiple-choice questionnaire. Only those who correctly answered all 10 questions proceeded with the consenting procedure. All volunteers gave a written informed consent before any study procedure was done. As EG is a Spanish-speaking country, approved Spanish-translated informed consent forms were used during the consenting process, which was conducted by investigators who were fluent in Spanish.

### Vaccine.

PfSPZ Vaccine is composed of aseptic, purified, metabolically active, nonreplicating (live, radiation attenuated) cryopreserved PfSPZ.^12,13^ The vaccine is stored in liquid nitrogen vapor phase at −150°C to −196°C. Before administration, PfSPZ Vaccine is thawed and then diluted in phosphate-buffered saline (PBS) with human serum albumin to achieve the correct dose.

### Study population and enrollment criteria.

Volunteers were healthy male adults between 18 and 35 years of age recruited from the towns of Baney and Rebola located in Baney District, Bioko Island. The study area has low malaria endemicity with estimated prevalence in 2016 of less than 1% in children aged 2 to 14 years. Volunteers were excluded from enrollment if they were taking immunosuppressive drugs, or had 1) clinically significant acute or chronic diseases, 2) clinically significant abnormalities in electrocardiogram at screening, 3) plans to travel outside Bioko Island, EG, in the first 9 months of the study, 4) immunosuppressive disorders, 5) a positive blood test for hepatitis B surface antigen (HBSAg), hepatitis C virus, or human immunodeficiency virus (HIV)-1, or 6) clinically significant abnormalities on basic hematology, hepatic and renal function laboratory tests. Volunteers with confirmed malaria parasitemia by microscopy of Giemsa-stained thick blood smears (sensitivity of 2–4 parasites/μL blood), intestinal parasites by microscopy of Lugol-stained fecal material (10× objective for detection and quantification and 40× objective for species identification), or urinary tract infections were treated and confirmed cured before they were enrolled into the study. In addition, volunteers with risk factors for clinically active tuberculosis plus a positive tuberculin skin test were excluded.

### Randomization and vaccination.

No randomization was performed in Group 1 in which all volunteers received PfSPZ Vaccine. Participants in Group 2 and 3 were randomized in a final ratio of 2:1 to receive three doses of either 2.7 × 10^5^ PfSPZ of PfSPZ Vaccine or NS. Randomization was done using a computer-generated list of random numbers by an independent statistician from the Swiss Tropical and Public Health Institute. At enrollment, volunteers were assigned either PfSPZ Vaccine or NS as specified in a master randomization list.

PfSPZ Vaccine and NS were administered as a 0.5 mL volume by DVI using a 1 mL syringe with a 25-gauge needle. Syringe preparation was done in a biological safety cabinet in a pharmacy out of sight of the study physicians and nurses who evaluated the study end points and of the study volunteers. Pharmacy personnel preparing the doses were aware of the treatment assignments, but took no other part in study-related procedures and were instructed not to reveal the assignments to either the volunteers or study investigators. The study clinical team was blinded to treatment.

### Definitions.

An AE was defined as any unfavorable or unintended change in body structure, body function or laboratory result associated temporally with the study treatment, whether or not it was considered to be related to the study treatment. A serious AE (SAE) was defined as any untoward medical occurrence that resulted in death, was life threatening, required hospitalization or prolongation of hospitalization, resulted in disability, or was otherwise considered serious by the investigators. All AEs were classified according to the preferred term in the Medical Dictionary for Regulatory Activities (MedDRA). When the report filed by the investigator did not clearly correspond to a MedDRA term, the most appropriate classification was clarified in discussion between the study sponsor and the investigators before unblinding.

### Outcome measures.

The primary outcome measures were the occurrence of solicited and unsolicited AEs during a 7-day and 28-day surveillance period after each vaccination, respectively. SAEs were collected throughout the study period, which extended 24 weeks after the last dose of study product. Local solicited AEs included erythema, swelling, induration, pain, and tenderness at the injection site. Systemic solicited AEs included malaise, nausea, vomiting, abdominal pain, diarrhea, fever (axillary temperature ≥ 37.5°C), arthralgia, myalgia, fatigue, chills, headache, chest pain/discomfort, shortness of breath, palpitations, and potential allergic reactions (wheezing, urticaria, edema/angioedema, hypotension, anaphylaxis). All solicited local AEs were considered definitely related to the study product and solicited systemic AEs were considered to be possibly or probably related because of existence of alternative causes.

### Safety monitoring.

Before being vaccinated, all volunteers were trained to measure their body temperature using digital thermometers. After each vaccination, the volunteers were evaluated for solicited AEs at 15 minutes, 1 hour, and 2 hours. After 2 hours, the volunteers were transported to their homes where study personnel collected GPS coordinates of their houses. These were kept in the volunteers’ files to facilitate follow-up in case of a missed scheduled visit. All volunteers were evaluated clinically at the study center 2, 7, 14, and 28 days after each vaccination, and every 4 weeks for 24 weeks after the last vaccination. In addition, study physicians called the volunteers by telephone on days 1, 3, 4, 5, and 6 after each vaccination to solicit local and systemic AEs. All volunteers were provided with mobile phones and encouraged to come to the study center or call the study physician when they felt unwell. Study physicians evaluated symptoms identified at scheduled visits and appropriate management was provided. In case further care was required, volunteers were referred to appropriate services at La Paz Medical Center where high-quality inpatient and outpatient care was available 24 hours a day, 7 days a week. Clinical evaluations consisted of measurement of vital signs and assessment for local injection site and general solicited signs or symptoms. Blood for safety analyses was collected at screening, before each vaccination and on days 2, 7, 14, and 28 after each vaccination, and every 4 weeks for 24 weeks after the last vaccination. At screening, a complete blood count and serum biochemistry (alanine aminotransferase [ALT] and aspartate aminotransferase [AST], creatinine, bilirubin, potassium, sodium, and alkaline phosphatase) were assessed. At subsequent scheduled visits, a complete blood count and limited biochemistry (ALT, AST, and creatinine) were assessed. Biochemistry tests were performed using a Pentra 60C+ and hematological tests were done with a Roche Cobas Integra 400 Plus.

Solicited local AEs were assessed as mild, moderate, severe, or life threatening (grades 1, 2, 3, and 4, respectively). Pain at the injection site was categorized as grade 1: does not interfere with activity, grade 2: interferes with activity or requires repeated use of non-narcotic pain reliever > 24 hour, grade 3: prevents daily activity or requires any use of narcotic pain reliever, or grade 4: requires hospitalization. Tenderness was graded as grade 1: mild discomfort to touch, grade 2: discomfort with movement, grade 3: significant discomfort at rest, or grade 4: discomfort that requires hospitalization. Erythema, swelling, or induration at the injection site measured at greatest single diameter were grade 1: > 2.5–5 cm, grade 2: 5.1–10 cm, grade 3: > 10 cm, or grade 4: presence of necrosis. Solicited systemic AEs were assessed as mild, moderate, severe, or life threatening (grades 1, 2, 3, and 4 respectively), defined as grade 1: AEs that were easily tolerated; grade 2: AEs that interfered with daily activity, grade 3: AEs that prevented daily activity, or grade 4: AEs that required hospitalization.

The degree to which an AE could be attributed to vaccination was determined by the principal investigator (PI) with advice from the rest of the clinical team and categorized as not, unlikely, possibly, probably, or definitely related. For the final analysis categories, “not related” and “unlikely related” were combined into “unrelated.” “Possibly related,” “probably related,” and “definitely related” were combined into “related.” Abnormal laboratory findings were graded using an FDA-recommended^14^ grading scheme adapted to be consistent with normal ranges in EG, Mali, and Tanzania. Abnormal laboratory tests were assessed as clinically significant if they were associated with clinical symptoms or required medical intervention or clinically nonsignificant if there were no associated symptoms, and they required no treatment. Information on any other symptoms, the use of any medications (prescription and/or over the counter), unscheduled medical consultations, and hospitalizations were also collected. All aspects of the trial were assessed by a sponsor-appointed clinical trial monitor, who also reviewed established safety stopping criteria together with the PI.

### Immunogenicity assessment.

Antibody responses were measured on sera obtained from participants before the first vaccination and 2 weeks after the last vaccination.

#### IgG antibodies to P. falciparum circumsporozoite protein (PfCSP) by enzyme-linked immunosorbent assay (ELISA).

Antibodies against PfCSP were measured by ELISA at Sanaria Inc. Briefly, 96-well plates (Thermo Fisher Scientific, Rochester, NY) were coated overnight at 4°C with 2.0 μg/mL per well of recombinant PfCSP in 50 μL coating buffer (KPL - Sera Care, Milford, MA). Plates were washed three times with 2 mM imidazole, 160 mM NaCl, 0.02% Tween 20, 0.5 mM EDTA, and then blocked with 1% bovine serum albumin (BSA), blocking buffer (KPL - Sera Care, Milford, MA) containing 1% nonfat dry milk for 1 hour at 37°C. The plates were washed three times and serially diluted serum samples (in triplicates) were added and incubated at 37°C for 1 hour. After three washes, peroxidase-labeled goat antihuman IgG (KPL - Sera Care, Milford, MA) was added at a dilution of 0.1 μg/mL and incubated at 37°C for 1 hour. Plates were washed three times, ABTS peroxidase substrate was added for plate development, and the plates were incubated for 75 minutes at 22°C. The plates were read with a Spectramax Plus384 microplate reader (Molecular Devices, Sunnyvale, CA) at 405 nm. The data were collected using Softmax Pro GXP v5 and fit to a 4-parameter logistic curve, to calculate the serum dilution at OD 1.0. A negative control (pooled serum from non-immune individuals from a malaria free area) was included in all assays. The positive control was pooled human sera taken 2 weeks after the last immunization from 12 volunteers immunized four or five times with PfSPZ Vaccine in the VRC 312 clinical trial, who did not develop parasitemia after CHMI.^6^ Samples were considered positive if the difference between the post-immunization OD 1.0 and the pre-immunization OD 1.0 (net OD 1.0) was ≥ 50, and the ratio of post-immunization OD 1.0 to pre-immunization OD 1.0 was ≥ 3.

#### IgG antibodies to PfSPZ by automated immunofluorescence assay (aIFA).

In the PfSPZ aIFA, the serum dilution at which the arbitrary fluorescence units (AFU) was 2 × 10^5^ was determined at Sanaria. Purified PfSPZ (NF54 strain) from aseptic *Anopheles stephensi* mosquitoes produced by Sanaria were resuspended in PBS (pH 7.4). Forty microliters containing 0.5 × 10^4^ PfSPZ were added to each well of Greiner cellstar clear bottom black 96-well plates (Greiner Bio-One GmbH, Frickenhausen, Germany), the plates were then left at ambient temperature for 12–18 hour to air-dry. Two-fold serial dilutions (50 μL/well) of sera, starting at 1:50, diluted in PBS with 2% BSA were added to each well of the 96-well plate containing air-dried PfSPZ and incubated at 37°C for 1 hour. The plates were washed in PBS three times using an Aquamax Microplate washer. Alexa Fluor 488–conjugated goat antihuman IgG (Life Technologies Corporation, Eugene, OR) was diluted 1:250 in PBS with 2% BSA, and 40 μL were added to each well. The plates were then incubated for 1 hour at 37°C, washed three times with PBS, and then 100 μL PBS was added to each well. The plates were sealed using a plate sealer and stored in the dark at 4°C until data acquisition. Samples were assessed by scanning the plates using an Acumen eX3 laser scanning imaging cytometer. The positive control was the same pooled human sera used for ELISA. The Acumen image cytometer scans the entire surface area of each well in a 96-well plate, and the fluorescence intensity values (arbitrary units) therefore represent the signal from all 0.5 × 10^4^ PfSPZ that were seeded in each well.

Data were plotted to fit a 4-parameter sigmoid curve in GraphPad Prism software using serum dilution (log transformed) as the *x* axis variable and AFU on the *y* axis. Over many iterations during development of this assay, we have determined that sera from naïve volunteers in the United States and Europe, including pre-immune sera, always register an arbitrary fluorescence value less than 2.0 × 10^5^ even at the highest concentration (1:50 dilution) used in this assay. Moreover, sera that do react to PfSPZ, 2.0 × 10^5^ AFU, fall in the exponential portion of their sigmoidal curves. Therefore, 2.0 × 10^5^ has been chosen as a threshold in the aIFA assay, and the results for each volunteer for antibodies to PfSPZ are reported as the reciprocal serum dilution at which fluorescence intensity was equal to 2.0 × 10^5^ AFU. For this study, the AFU values were calculated for sera collected before immunization and after immunization because individuals pre-exposed to malaria in EG were expected to harbor antibodies to PfSPZs before immunization with PfSPZ Vaccine. Sera were considered positive for seroconversion if their net AFU 2 × 10^5^ and AFU 2 × 10^5^ ratio, calculated respectively by subtracting the pre-vaccination from the post-vaccination AFU 2 × 10^5^ and dividing the post-vaccination by the pre-vaccination AFU 2 × 10^5^ were ≥ 150 and ≥ 3.0, respectively.

#### Functional antibodies to PfSPZ by inhibition of sporozoite invasion (ISI) assay.

HC-04 (1F9) cells (hepatocytes) were cultured in complete medium (10% FBS in DMEM/F12 with 100 units/mL penicillin and 100 μg/mL streptomycin; Gibco by Life Technologies, Grand Island, NY) in (Entactin-Collagen IV-Laminin) ECL-coated 96-well clear bottom black well plates (Greiner Bio-One GmbH, Frickenhausen, Germany) at a density of 2.5 × 10^4^ cells per well, and incubated for 24 hours at 37°C, 5% CO_2_ with 85% relative humidity. Twenty-four hours later, cells were infected with 10^4^ aseptic, purified, cryopreserved PfSPZ per well, without or with sera diluted in an 11-point dilution series from subjects immunized with PfSPZ Vaccine. The assay control included PfSPZ added with media alone. Sera were assessed at pre-immunization (baseline) and predetermined time points after immunization. PfSPZ that had not invaded the HC-04 cells were removed 3 hours later by washing three times with Dulbecco’s phosphate-buffered saline, and the cultures were fixed using 4% paraformaldehyde for 15 minutes at room temperature. Differential immunostaining was performed to distinguish between PfSPZ inside the hepatocytes versus PfSPZ outside the hepatocytes. PfSPZ outside the hepatocytes were stained with an anti-PfCSP mAb (2A10, 6.86 μg/mL) (Protein Potential LLC, with permission from New York University School of Medicine) conjugated with Alexa Fluor 633 (far-red) (1 μg/mL; custom-conjugated at GenScript^®^ USA Inc., Piscataway, NJ), a 1:500 dilution. The hepatocytes were then permeabilized with 0.1% Triton X-100 for 15 minutes at room temperature, and the PfSPZ inside the hepatocytes were stained with the anti-PfCSP mAb (2A10, 6.86 μg/mL) conjugated with Alexa Fluor 488 (green; 1 μg/mL, conjugated from Genscript USA Inc., Piscataway, NJ), a 1:500 dilution.

The numbers and intensity of infected hepatocytes (green only) were counted by scanning the plates using an Acumen eX3 laser scanning imaging cytometer. Inhibitory activity at each serum dilution sera was calculated using the following formula:

$$\text{Percent}\;\text{inhibition}=100-\left(\frac{\text{Fluorescence}\;\text{values}\;\text{of}\;\text{invaded}\;\text{PfSPZ}\;\text{in}\;\text{presence}\;\text{of}\;\text{serum*}100}{\text{Fluorescence}\;\text{values}\;\text{of}\;\text{invaded}\;\text{PfSPZ}\;\text{in}\;\text{media}\;\text{control}}\right)$$

The number of invaded PfSPZ scored in this assay in the absence of serum was 400–600, giving an intensity of 1–3 × 10^6^ fluorescence units. Data were plotted to fit a 4-parameter sigmoid curve in GraphPad Prism software using serum dilution (log transformed) as the *x* axis variable and percent inhibition on the *y* axis. Eighty percent inhibition was interpolated from the sigmoidal curves as the reciprocal serum dilution at which the fluorescence intensity of infected wells with serum was 20% of the negative control without serum. The serum dilution at which inhibition of PfSPZ invasion into hepatocytes was 80% as compared with a negative control without serum was designated as ISI activity. Sera with a net ISI activity of ≥ 10% between post- and pre-immunization sera and a ratio of post- to pre-vaccination ISI activity of ≥ 3.0 were considered positive.

### Statistical analysis.

This was a phase 1 clinical trial with no formal sample size calculation. We compared the proportion of subjects with AEs as well as frequencies of individual AEs between vaccination groups recorded from the first vaccination through study conclusion (24 weeks post last vaccination).

The data for local and systemic AEs were grouped by vaccination groups and summarized by frequency and severity of AEs using descriptive statistics. A two-sided Fisher’s exact test was used to compare the frequency of AEs between vaccinees and controls. For the immunogenicity analysis, change from the baseline of anti-PfCSP antibodies was plotted by vaccine group and time points together with the median and interquartile ranges. Nonparametric statistics (Wilcoxon–Mann–Whitney test) were used to compare the change of PfSPZ antibody from the baseline between PfSPZ Vaccine and NS groups. The Spearman correlation coefficient was used to assess the association between the results of the different antibody assays. Fixed effect regression model was used to evaluate the effect of PfSPZ Vaccine on hematology (white blood cells [WBCs], neutrophils, and eosinophils) parameters taking into account the repeated measurements in each volunteer over time. Data were analyzed using STATA 13.0. Anti-PfCSP antibody activities were compared between different clinical trials using a Kruskal–Wallis test followed by a Dunn’s multiple comparisons test for significance between pairs. These data were analyzed using GraphPad Prism 7.

### Genotyping of parasites.

One volunteer developed asymptomatic Pf infection 5 months after the third dose of PfSPZ Vaccine. The Pf parasites from this volunteer were assessed by microsatellite analysis^15^ to determine if the parasites were derived from PfSPZ Vaccine (PfNF54) or from a naturally acquired infection. The DNA was isolated from a blood specimen using Quick-gDNA Blood MiniPrep Kit (Zymo Research, Irvine, CA). Unlinked microsatellite markers Poly alpha, PfPK2, TA81, ARA2, TA87, and TA40 were amplified using heminested PCR.^15^ Capillary electrophoresis was performed using an Applied Biosystems 3730XL 96-capillary DNA sequencer and software. Capillary electrophoresis output files were analyzed using Geneious 8.1.8 software. The detailed protocol is available on the University of Maryland website (http://www.medschool.umaryland.edu/malaria/Protocols/). Genomic control strains NF54, 3D7, 7G8, HB3, and V1S (ATCC-MR4, Manassas, VA) were included to determine characteristic peak morphology for each microsatellite locus and control for slight variations among runs. Peak sizes were determined by manual inspection of each electropherogram and then normalized against the Genescan^™^ 600 Liz^®^ size standard (Applied Biosystems, Foster City, CA). Normalized peak sizes were compared with those observed for PfNF54.

## RESULTS

### Study population.

**Figure 1.**
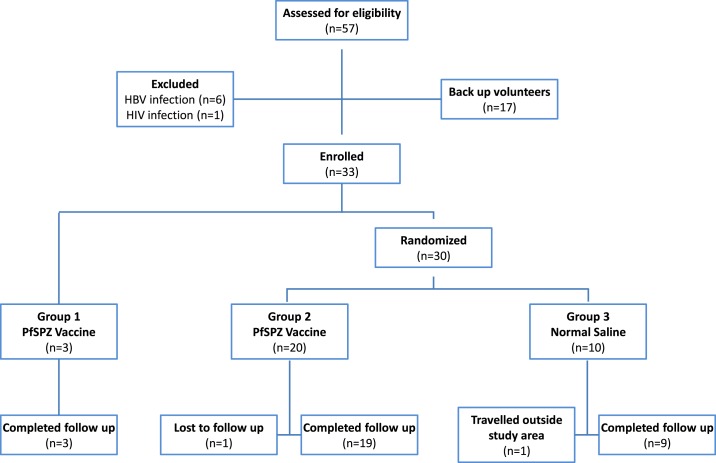
Consort diagram for EGSPZV1 clinical trial.

Between March and April 2015, 57 adults were screened and of the 40 who met the eligibility criteria, 33 were enrolled into the study (Figure 1). Three volunteers were enrolled into Group 1, and 30 volunteers were randomized into Groups 2 (*N* = 20) and 3 (*N* = 10). The most common reasons for exclusion were positive HBSAg and/or positive HIV tests. Of 57 volunteers screened, helminth infections were diagnosed in 31 (54%). The mean age at enrollment was 20 (17–26) years in the PfSPZ Vaccine group and 23 (18–33) years in the control group. All the groups were generally similar at enrollment with regard to age and body mass index (Table 1). Of the 33 volunteers, all received all scheduled immunizations, and 31 (94%) completed all follow-up visits. Two volunteers failed to complete their follow-up; one vaccinee traveled abroad for further studies 2 months after the last vaccination, and one control was lost to follow up at 5 months after the last vaccination. The two volunteers were healthy at their last scheduled visits.

**Table 1:** Demographic and baseline characteristics by group of study population

	Group 1 PfSPZ Vaccine	Group 2 PfSPZ Vaccine	Group 3 normal saline
Number of volunteers	3	20	10
Mean age (range)	19.7 (19–20)	20.0 (17–26)	22.9 (18–33)
Mean body mass index (range)	22.5 (20.6–24.8)	23.0 (18.8–28.6)	22.1 (19.5–24.0)

PfSPZ = *Plasmodium falciparum* sporozoites.

### Vaccine administration.

Vaccinations took place over a 4-month period from March through August 2015. Study nurses administered the vaccine by DVI. Injection by DVI was completed in one attempt in 88 of 96 (92%) administrations. The other eight administrations were completed in two attempts. The nurse performing the injection judged the procedure as simple in 87 of the 88 (99%) single-attempt injections. Of 96 administrations, 91 (95%) were judged to be painless by the volunteers, and five (5%) were judged to be associated with mild pain. The entire DVI procedure from the time of syringe handover to completion of injection took a mean of 3.80 (1–8) minutes, 2.19 (1–5) minutes, and 2.40 (1–5) minutes in the safety sentinel, PfSPZ Vaccine, and NS groups, respectively.

### Solicited and unsolicited AEs.

**Figure 2.**
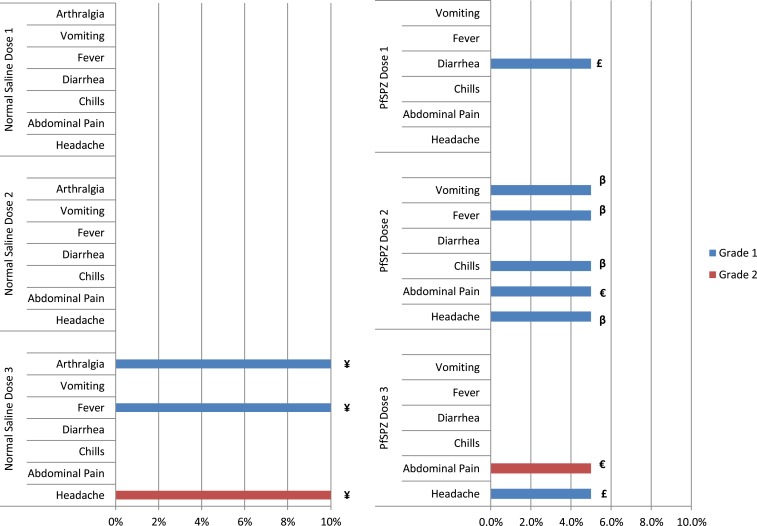
Comparison of adverse events (AEs) in volunteers receiving normal saline (NS) and PfSPZ Vaccine. The percent of volunteers with a specific AE is depicted. Only a single volunteer had any individual AE after each immunization (maximum of 1/10 [10%] for NS and 1/20 [5%] for PfSPZ Vaccine). ¥, €, £, β are individual volunteers. This figure appears in color at www.ajtmh.org.

No volunteer developed local or systemic solicited AEs in the sentinel group of three volunteers who received two vaccinations with PfSPZ Vaccine. No local solicited AEs were reported after administration of any of the three doses of PfSPZ Vaccine and NS in the main groups. Overall, three of 20 (15%) and one of 10 (10%) volunteers reported a systemic solicited AE after any of the three doses in the PfSPZ Vaccine and NS groups, respectively (Figure 2). Of the 11 reported systemic AEs, nine (82%) were grade 1, and 2 (18%) were grade 2 (one in the PfSPZ Vaccine and one in the NS group) (Figure 2). All solicited AEs resolved without sequelae within 7 days after vaccination. There was no evidence of increasing frequency or severity of reported local and/or systemic solicited AEs with subsequent doses of vaccine nor were there reports of anaphylaxis or any other serious allergic reactions. There was no significant difference in the frequency of solicited AEs between PfSPZ Vaccine and NS groups (Table 2).

**Table 2:** Number of solicited AEs after an injection in recipients of NS and PfSPZ Vaccine

AE	NS *N* (%)*	PfSPZ Vaccine *N* (%)*
Local (site of injection)		
Any AE	0	0
Systemic		
Headache	1 (3%)	2 (3%)
Abdominal pain	0	2 (3%)
Chills	0	1 (2%)
Diarrhea	0	1 (2%)
Vomiting	0	1 (2%)
Arthralgia	1 (3%)	0
Fever	1 (3%)	1 (2%)
Myalgia	0	0
Allergic reactions	0	0
Chest pain/palpitations/shortness of breath	0	0
TOTAL	3 (10%)	8 (13%)

AE = adverse event; N = number of AEs; NS = normal saline; PfSPZ = *Plasmodium falciparum* sporozoites.

% = Percentage of injections which gave rise to the AE using number of injections (60 injections for vaccine group, 30 injections for NS group) as denominator. There were no significant differences in the incidence of solicited AEs between recipients of NS and PfSPZ Vaccine.

* The *P* value for the Fisher exact test (2-sided) comparing the two groups for each AE and overall AE rate was > 0.05 for all comparisons.

There were 32 unsolicited AEs reported during the study, of which 27 (84.4%) were mild (grade 1), three (9.4%) were moderate (grade 2), and two (6.2%) were of severe (grade 3) severity (Table 3). The two severe unsolicited AEs were periodontitis and toothache. One volunteer completed treatment of periodontitis and recovered within 16 days. The other volunteer’s severe toothache resolved after 2 days. Subsequent evaluation revealed that he had pre-existing dental caries. None of the unsolicited AEs were considered related to the Investigational Product. All other unsolicited AEs resolved without sequelae. There was no statistically significant difference in frequency of unsolicited AEs between volunteers receiving PfSPZ Vaccine and those receiving NS (*P* = 0.319, Fisher’s exact test, 2-tailed) (Table 3).

**Table 3:** Number of unsolicited AEs in recipients of NS and PfSPZ Vaccine

Unsolicited AEs (MedRA term)	NS *N* (%)*	PfSPZ Vaccine *N* (%)*
Dental and periodontal infections	1 (6.3%)	3 (18.8%)
Ear and labyrinth disorders	0 (0.0%)	1 (6.3%)
Gastrointestinal disorders	0 (0.0%)	1 (6.3%)
General disorders	3 (18.8%)	1 (6.3%)
Infections and infestations	6 (37.5%)	8 (50.0%)
Injury and procedural complications	2 (12.5%)	0 (0.0%)
Musculoskeletal and connective tissue disorders	3 (18.8%)	1 (6.3%)
Nervous system disorders	0 (0.0%)	1 (6.3%)
Skin and subcutaneous tissue disorders	1 (6.3%)	0 (0.0%)
Total number of unsolicited AEs	16	16

AE = adverse event; NS = normal saline; PfSPZ = *Plasmodium falciparum* sporozoites. There were no significant differences in the incidence of unsolicited AEs between recipients of NS and PfSPZ Vaccine. The *P* values were calculated on a per volunteer basis (e.g. 3/20 vs. 1/10 for dental and periodontal infections) in a two-sided Fisher’s exact test. All *P* values were ≥ 0.1.

* The numbers in brackets are calculated as the proportion of the total unsolicited AEs reported.

One volunteer developed asymptomatic Pf infection 5 months after the third dose of PfSPZ Vaccine. The volunteer was treated with artesunate–amodiaquine, and a repeat blood smear 7 days later confirmed clearance of parasitemia. The microsatellite patterns of the DNA from the parasites from this individual indicated that the volunteer was not infected with PfNF54, the Pf strain used to make PfSPZ Vaccine. The microsatellite analysis indicated that the volunteer was infected with multiple other strains of Pf.

Overall, no unexpected or SAEs occurred. Furthermore, no volunteers were withdrawn because of AEs.

### Laboratory safety tests.

Most of the abnormal hematology (WBC and differentials) and biochemistry (serum creatinine, ALT and AST) laboratory abnormalities were grade 1 and 2 (Table 4). Eosinophilia was a common laboratory abnormality, occurring at some point in most volunteers and often associated with the presence of helminth infections, which included *Ascaris lumbricoides*, *Trichuris trichiura*, and *Schistosoma haematobium*. The only grade 3 laboratory abnormality identified after vaccination in the main study groups, an elevated eosinophil count in a PfSPZ Vaccine recipient, was associated with infection with *S. haematobium*, and fell to grade 1 after treatment. Transient, grade 1–2 decreases in neutrophil counts were also common, but showed no relationship to administration of PfSPZ Vaccine, occurring more frequently in control volunteers than vaccine recipients. Eosinophils and WBCs had a minimal, but significant downward trend during the study period in the PfSPZ Vaccine group, but not in NS controls (data not shown). Elevated ALT and AST levels, which could theoretically be expected after the administration of a live PfSPZ vaccine, occurred with similar frequency in vaccine and control volunteers. In summary, there were no significant trends or group differences in laboratory parameters indicating a vaccine effect.

**Table 4:** Laboratory abnormalities

Laboratory parameter*	Grade abnormality	Pilot *N* (%)		PfSPZ Vaccine *N* (%)		Normal saline *N* (%)	
Decreased hemoglobin	1	0	(0%)	0	(0%)	1	(10%)
Decreased platelets	1	0	(0%)	4	(20%)	0	(0%)
Increased WBC count	1	0	(0%)	5	(25%)	2	(20%)
	2	–	–	1	(5%)	–	–
Decreased WBC count	1	1	(33%)	5	(25%)	5	(50%
Decreased neutrophils	1	1	(33%)	17	(85%)	8	(80%)
	2	–	–	1	(5%)	2	(20%)
Decreased lymphocytes	1	1	(33%)	0	(0%)	1	(10%)
Increased eosinophils	1	0	(0%)	14	(70%)	10	(100%)
	2	–	–	11	(55%)	3	(30%)
	3	–	–	1	(5%)	–	–
Elevated ALT	1	1	(33%)	8	(40%)	4	(40%)
Elevated AST	1	0	(0%)	6	(30%)	3	(30%)
	2	–	–	2	(10%)	–	–
Elevated total bilirubin	1	0	(0%)	0	(0%)	0	(0%)
Elevated creatinine	1	1	(33%)	0	(0%)	2	(20%)
Hypoglycemia	1	0	(0%)	2	(10%)	0	(0%)
Hyperglycemia	1	1	(33%)	2	(10%)	1	(10%)
	2	1	(33%)	–	–	1	(10%)

ALT = alanine aminotransferase; AST = aspartate aminotransferase; N = number of volunteers experiencing the abnormality; PfSPZ = *Plasmodium falciparum* sporozoites; WBC = white blood cell; % = proportion of the number of volunteers in the group experiencing the abnormality. Number (percent) of volunteers experiencing laboratory abnormalities after vaccination (excluding abnormalities identified at screening or prior to the first vaccination).

* Abnormal Laboratory Values are defined as grade 1 or higher per the protocol defined toxicity ranges.

### Immunogenicity.

**Figure 3.**
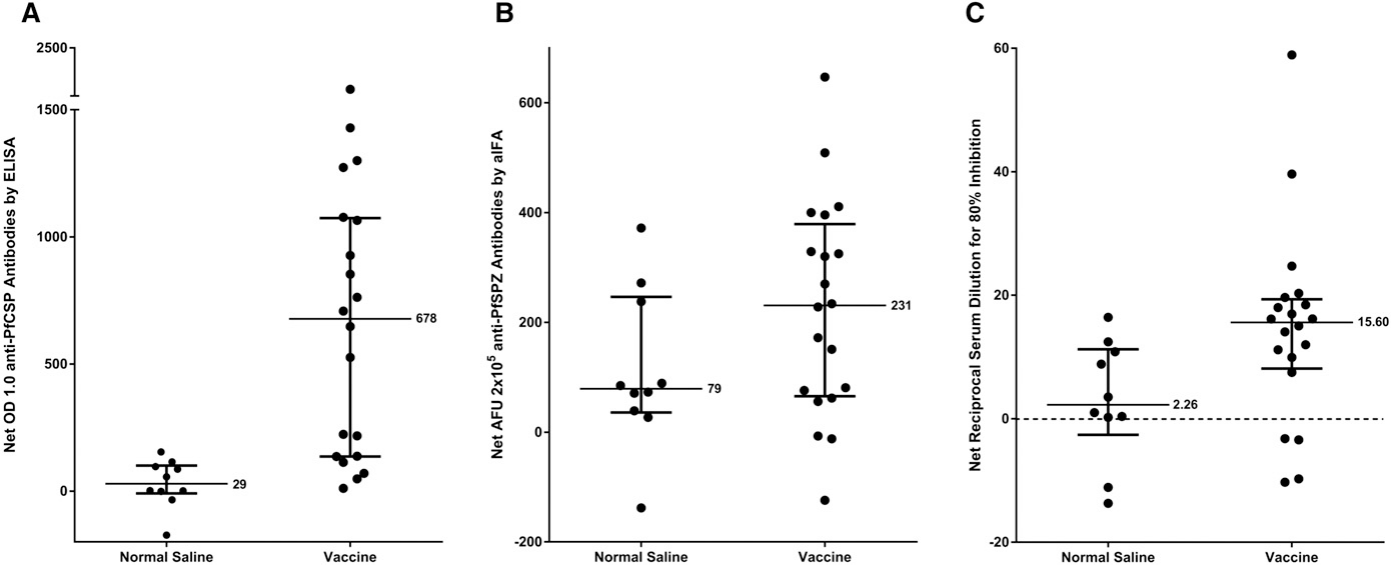
Antibody responses 2 weeks after the last vaccination. Responses in all vaccinees and controls who received three doses of PfSPZ Vaccine or normal saline were measured by (**A**) *Plasmodium falciparum* (Pf) circumsporozoite protein (PfCSP) enzyme-linked immunosorbent assay (ELISA), (**B**) Automated immunofluorescence assay (aIFA), and (**C**) Inhibition of sporozoite invasion assay (ISI) assay. In the ELISA, antibody responses are reported as the net OD 1.0. OD 1.0 is the serum dilution at which the optical density is 1.0, and net OD 1.0 is the difference between the post- and pre-immunization OD 1.0. In the aIFA assay, antibody responses are reported as the reciprocal serum dilution at which the arbitrary fluorescence units (AFU) were 2 × 10^5^. In the ISI assay, antibody responses are reported as the reciprocal serum dilution that gave 80% inhibition of PfSPZ invasion. Medians with interquartile ranges are shown.

In the PfCSP ELISA, 14/20 (70%) of vaccinated subjects and 0/10 (0%) of control subjects were considered to have become positive as compared with before immunization (*P* = 0.0003, Fisher’s exact test, 2-tailed) with a median net OD 1.0 of positives of 890 and the median OD 1.0 ratio of positives of 9.50 (Table 5). Among positives, the net OD 1.0 ranged from 136 to 2,414, and the OD 1.0 ratio ranged from 3.10 to 23.66 (Figure 3A) (Table 5).

**Table 5:** Immune responses in volunteers receiving NS and PfSPZ Vaccine before the first immunization and 2 weeks after the third immunization

Group	Volunteer ID	ELISA PfCSP OD 1.0				aIFA AFU 2 × 10^5^				ISI reciprocal serum dilution for 80% inhibition			
		Pre-immune*	Immune†	Net‡	Ratio§	Pre-immune*	Immune†	Net‡	Ratio§	Pre-immune*	Immune†	Net‡	Ratio§
NS	201	188	342	154	1.82	0	73	73	73.00	**4.91**	**21.33**	**16.41**	**4.34**
	222	192	278	86	1.45	0	71	71	71.00	15.31	16.31	0.99	1.06
	225	38	37	−1	0.97	**73**	**345**	**272**	**4.73**	13.72	2.60	−11.13	0.19
	232	102	158	56	1.55	54	81	27	1.50	0.00	8.83	8.83	8.83
	235	62	158	96	2.55	127	216	89	1.70	13.76	14.11	0.36	1.03
	241	167	133	−34	0.80	0	39	39	39.00	9.36	12.88	3.52	1.38
	251	654	481	−173	0.74	138	0	−138	0.00	17.43	3.75	−13.69	0.21
	253	45	47	2	1.04	19	104	85	5.47	10.17	21.02	10.84	2.07
	256	95	210	115	2.21	**0**	**238**	**238**	**238.00**	13.15	25.61	12.46	1.95
	258	39	40	1	1.03	**0**	**372**	**372**	**372.00**	7.31	7.55	0.23	1.03
	Median	99	158	29	1.25	19	93	79	22.24	11.66	13.50	2.26	1.22
PfSPZ Vaccine	203	**46**	**808**	**762**	**17.57**	**0**	**270**	**270**	**270.00**	**0.00**	**9.93**	**9.93**	**9.93**
	204	328	551	223	1.68	134	122	−12	0.91	12.51	28.69	16.18	2.29
	206	275	412	137	1.50	212	268	56	1.26	16.05	5.75	−10.29	0.36
	212	**172**	**1,472**	**1,300**	**8.56**	**18**	**252**	**234**	**14.00**	**8.60**	**67.53**	**58.93**	**7.85**
	215	**77**	**603**	**526**	**7.83**	**88**	**488**	**400**	**5.55**	**0.00**	**14.06**	**14.06**	**14.06**
	217	**85**	**938**	**853**	**11.04**	**74**	**470**	**396**	**6.35**	**4.55**	**20.71**	**16.15**	**4.55**
	218	**89**	**736**	**647**	**8.27**	**82**	**254**	**172**	**3.10**	13.55	31.99	18.44	2.36
	226	311	381	70	1.23	259	340	81	1.31	5.65	2.44	−3.22	0.43
	227	525	536	11	1.02	417	293	−124	0.70	12.67	27.71	15.04	2.19
	229	**237**	**1,314**	**1,077**	**5.54**	**127**	**538**	**411**	**4.24**	21.80	33.79	11.99	1.55
	231	**118**	**1,547**	**1,429**	**13.11**	**84**	**404**	**320**	**4.81**	**0.00**	**39.64**	**39.64**	**39.64**
	233	**199**	**1,472**	**1,273**	**7.40**	**59**	**706**	**647**	**11.97**	**0.00**	**20.32**	**20.32**	**20.32**
	236	**62**	**198**	**136**	**3.19**	299	292	−7	0.98	**3.07**	**20.03**	**16.96**	**6.52**
	242	**47**	**755**	**708**	**16.06**	**16**	**341**	**325**	**21.31**	21.38	39.39	18.01	1.84
	243	**188**	**1,115**	**927**	**5.93**	**0**	**329**	**329**	**329.00**	15.27	5.53	−9.74	0.36
	244	**23**	**240**	**217**	**10.43**	**0**	**151**	**151**	**151.00**	**0.00**	**11.18**	**11.18**	**11.18**
	249	106	219	113	2.07	71	147	76	2.07	10.95	18.46	7.51	1.69
	250	**47**	**1,112**	**1,065**	**23.66**	**3**	**512**	**509**	**170.67**	21.35	46.07	24.72	2.16
	252	**236**	**2,650**	**2,414**	**11.23**	**44**	**272**	**228**	**6.18**	**8.13**	**27.76**	**19.63**	**3.41**
	257	42	90	48	2.14	45	107	62	2.38	22.56	19.12	−3.43	0.85
	Median	112	746	678	7.61	72	292	231	5.18	9.77	20.51	15.60	2.33

AFU = arbitrary fluorescence units; aIFA = automated immunofluorescence assay; ELISA = enzyme-linked immunosorbent assay; ISI = inhibition of sporozoite invasion assay; NS = normal saline; PfCSP = *Plasmodium falciparum* circumsporozoite protein; PfSPZ = *P. falciparum* sporozoites; Volunteer ID = volunteer identification number. Immune responses were measured in all Group 2 and Group 3 volunteers using PfCSP ELISA, aIFA, and ISI assays. Those in bold were considered to have developed antibodies after immunization.

* Pre-immune sera taken before the first immunization.

† Immune sera taken 2 weeks after the last immunization.

‡ Net = immune–pre-immune value.

§ Ratio = immune/pre-immune value. All values of 0 were changed to 1 when calculating ratios.

In the aIFA, 13/20 (65%) of vaccinated subjects and 3/10 (30%) of control subjects were considered to have become positive as compared with before immunization (Figure 3B) (Table 5) (*P* = 0.12). Among positive vaccinees, the net AFU 2 × 10^5^ ranged from 150 to 647, and the AFU 2 × 10^5^ ratio ranged from 3.10 to 270 (Table 5). Of the 14 vaccinees who seroconverted by PfCSP ELISA, only one did not seroconvert by PfSPZ aIFA (Table 5).

In the ISI, 9/20 vaccinees and 1/10 control subjects were considered to have become positive as compared with before immunization (Figure 3C) (Table 5) (*P* = 0.10). All nine were also positive by PfCSP ELISA, and 8/9 were positive by the aIFA. Among positives, the net ISI activity ranged from 11.42 to 63.23 and the ISI activity ratio ranged from 3.14 to 25.63 (Table 5).

The antibody responses by ELISA (net OD 1.0) and net ISI activity (80% inhibition of PfSPZ invasion) were positively correlated 2 weeks after the third dose (*r*^2^ = 0.39, *P* = 0.0033); the same was true for correlation between PfCSP ELISA and net aIFA (*r*^2^ = 0.52, *P* = 0.0003). There was no significant correlation between net aIFA and net ISI (*r*^2^ = 0.11, *P* = 0.16).

**Figure 4.**
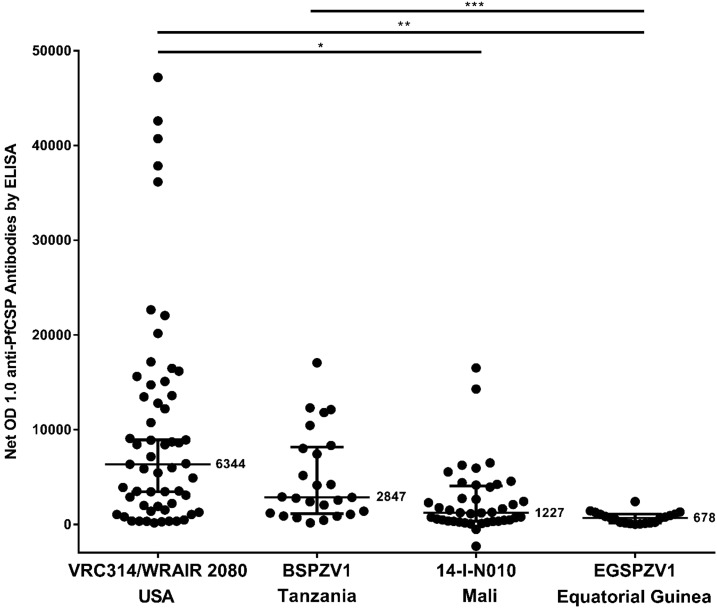
Comparison of anti-*Plasmodium falciparum* circumsporozoite protein (PfCSP) antibody responses in four different clinical trials in which the same dose of PfSPZ Vaccine was administered. Antibody responses measured by enzyme-linked immunosorbent assay (ELISA) are reported as the net OD 1.0. The OD 1.0 is the serum dilution at which the optical density is 1.0, and net OD 1.0 is the difference between the post- and pre-immunization OD 1.0. In the USA (VRC314/WRAIR 2080) (most subjects), Mali and Tanzania vaccinations were at 0, 4, and 8 weeks. In Equatorial Guinea (EG), vaccinations were at 0, 8, and 16 weeks. In the United States, Mali, and EG, sera were drawn at 2 weeks after the last vaccination. In Tanzania, sera were drawn at 4 weeks after the last vaccination. Bars with asterisks indicate the statistical significance, as determined by a Kruskal–Wallis test followed by a Dunn’s multiple comparisons test (*P* = * < 0.0001; ** < 0.0001; *** 0.0008).

We compared the anti-PfCSP responses by ELISA at 2 weeks (United States, Mali, EG) or 4 weeks (Tanzania) after administration of three doses at 8-week intervals of 2.7 × 10^5^ PfSPZ to adults in EG, to responses to three doses of 2.7 × 10^5^ PfSPZ administered to adults at 4-week intervals in Mali^7^ and Tanzania (Jongo, unpublished data), and in the United States at 4-week intervals or over 20 weeks^8,9^ (Figure 4). Responses were significantly lower in Mali and EG than in the United States, and the responses in EG were significantly lower than in Tanzanian young adults who had minimal exposure to malaria during the previous 5 years. There was no difference in responses in sera from Mali and EG.

## DISCUSSION

We have conducted the first clinical trial in the history of EG. There were significant regulatory, quality, clinical, and logistical challenges in conducting this trial fully adhering to the international standards of Good Clinical Practices. A detailed report of these developments and capacity building efforts will be published elsewhere. The trial was successful in showing that three doses of 2.7 × 10^5^ PfSPZ of PfSPZ Vaccine were safe, well tolerated, and moderately immunogenic in healthy 18–35-year-old male Equatoguineans living in an area with low malaria exposure.

Almost all the vaccinees and controls indicated that there was no pain associated with injection of PfSPZ Vaccine by DVI. There were no differences in the rate of solicited or unsolicited AEs between the 20 volunteers who received PfSPZ Vaccine and the 10 who received NS. There were no local solicited AEs or severe solicited systemic AEs after vaccination, and only two grade 3 unsolicited AEs were reported during the trial, both unrelated to investigational product. We observed no unexpected trends in hematological (hemoglobin, hematocrit, WBCs, platelets) or biochemical (creatinine, ALT, AST) laboratory markers except that eosinophils and WBCs had a minimal, but significant downward trend during the study period in the PfSPZ Vaccine group, but not in NS controls. We have no explanation for this finding. The excellent reactogenicity and safety profile in Equatoguineans immunized with PfSPZ Vaccine is consistent with findings from Mali^7^ and Tanzania (Jongo et al., unpublished data) where similar doses of PfSPZ Vaccine were administered. Higher doses of PfSPZ Vaccine are currently being assessed in infants, children, and adults in EG.

The tolerability and safety profiles are also consistent with results of multiple other studies of PfSPZ Vaccine,^6–10,13^ PfSPZ Challenge (live PfSPZ for infection),^16–19^ and PfSPZ-CVac (PfSPZ Challenge with an antimalarial drug).^20,21^ So far, no breakthrough malaria infections have been observed with Sanaria’s radiation-attenuated PfSPZ Vaccine. There was no evidence for local or severe systemic AEs, thought to be due to the quality (e.g., purity and sterility) of PfSPZ Vaccine. This favorable safety and tolerability profile will facilitate compliance if PfSPZ Vaccine is used, as planned,^22,23^ in mass vaccination programs.

PfSPZ Vaccine induced antibody responses to PfCSP and PfSPZ. However, induction of functional activity assessed by ISI was modest. The antibody responses in this EG study were significantly lower than in a U.S. study in which the same immunization regimen was administered, but similar to antibody responses in a study in a highly endemic area of Mali (Figure 4). Interestingly, antibody responses were higher in young adult Tanzanians who were selected for having had minimal exposure to malaria during the past 5 years (Figure 4). We hypothesize that long-term exposure to Pf infections leads to downregulation of immune responses to PfSPZ Vaccine. It is also possible that an activated immune microenvironment, compared with that in Americans, reduced the antibody responses. This has been shown for a yellow fever vaccine study in Entebbe, Uganda.^24^ Additional studies will be needed to better explain and overcome this poor immunogenicity.

The baseline rate of helminth infection (54%) was high. The most common helminths were *A. lumbricoides*, *T. trichiura* and *S. haematobium*. Helminth infection can impair responses to vaccines against various diseases,^25–27^ and specifically impaired antibody responses after vaccination with subunit malaria vaccines in humans^28^ and mice.^29,30^ In contrast, helminth infection did not affect the immunogenicity and protection of radiation-attenuated SPZ vaccine in mice.^31^ The helminth rate at enrollment was significantly lower in Mali (3/93 (3.2%)^7^ where antibody responses were similarly low (Figure 4). Thus, we do not think that active, ongoing helminth infections alone were responsible for the poor antibody responses. Nonetheless, we will continue to study the impact of helminth infections on immunogenicity and protective efficacy where possible.

Trained study nurses performed all of the DVI injections of PfSPZ Vaccine and NS. The procedure took little time to learn or to perform and was largely painless. At the time of writing this manuscript, > 4,000 injections by DVI of PfSPZ Vaccine, PfSPZ-CVac,^20,21^ PfSPZ Challenge,^16–19^ or NS placebo have been administered to > 1,900 individuals worldwide, including infants and children in Tanzania, Kenya, and EG. It is more challenging to administer by DVI to children less than 2 years of age than to older children, adolescents, and adults. However, experience from studies conducted in Mali and Tanzania shows that injection skills improve significantly with practice, and more than 350 5–12-month-old infants have successfully received PfSPZ Vaccine or NS placebo by DVI. Data from an ongoing PfSPZ Vaccine study in Kenya including more than 300 infants will provide even more insight into the operational requirements for DVI in young children and infants.

In conclusion, PfSPZ Vaccine was safe and well tolerated in young Equatoguinean adults living in an area of low malaria transmission, but the vaccine induced lower anti-PfCSP antibody responses compared with those in malaria-naïve adults. Further studies are ongoing to improve the immunogenicity through dose escalation. By successfully conducting the first clinical trial in the country’s history, we have laid the foundation for development of a robust research and development program in EG that will contribute to future malaria elimination efforts.
